# Ling-Ling Chen: RNA has its own features; don’t study it as a protein

**DOI:** 10.1093/nsr/nwad287

**Published:** 2023-11-20

**Authors:** Weijie Zhao

**Affiliations:** NSR news editor based in Beijing, China

## Abstract

Non-coding RNA (ncRNA) has been a very active research area over the past 30 years. From small ncRNA – the discovery of RNA interference won the lead researchers the Nobel Prize, to long ncRNA (lncRNA), which has attracted much attention in recent years, various ncRNAs participate in all kinds of biological processes and show a variety of biomedical application prospects. Recently, *National Science Review* (*NSR*) interviewed Ling-Ling Chen, a professor at the Center for Excellence in Molecular Cell Science (CEMCS) of the Chinese Academy of Sciences (CAS), deputy director of the State Key Laboratory of Molecular Biology and director of the CAS Key Laboratory of RNA Science and Engineering, to talk about the magic world of RNA.

Ling-Ling Chen and her team have been studying ncRNA for more than 10 years, and have witnessed and promoted the development of this field. They discovered unconventional lncRNAs without polyadenylated (polyA) tails or N7-methylguanosine (m^7^G) caps, including sno-lncRNAs (small nucleolar lncRNAs), SPAs (5' snoRNA capped and 3' polyadenylated RNAs) and circRNAs (circular RNAs), and have made remarkable progress clarifying the biogenetic mechanisms and functions of these RNAs and exploring their biomedical application. Chen said: ‘I am 45 years old. I hope I will not stagnate here, but make new discoveries.’

## Teaser

An Interview with Ling-Ling Chen about non-coding RNAs.

## THE LARGE FAMILY OF RNAS: FAR BEYOND PROTEIN-CODING

***NSR*: What are the major types of ncRNAs? What is the history of research in this field?**

**Chen:** ncRNA research can be divided into several stages. The first stage was from the 1960s to the 1980s. During this period, housekeeping ncRNAs were discovered, such as ribosomal RNA, transfer RNA, small nuclear RNA, small nucleolar RNA and so on. These housekeeping ncRNAs are abundant in cells and have essential physiological functions.

The second stage was from the early 1990s to around 2005, during which a series of small ncRNAs were discovered, including microRNAs that can silence gene expression, piRNAs (PIWI-interacting RNAs) related to mammalian reproduction and so on. Functional studies have shown that these small ncRNAs can be used in gene expression regulation, crop breeding, gene therapy and many other fields. In fact, to date, the study of small RNA has not stopped. In 2006, two scientists, Andrew Fire and Craig Mello, won the Nobel Prize in Physiology or Medicine for their discovery of RNA interference. In 2018, the US Food and Drug Administration approved the first siRNA (small interfering RNA) drug to treat nerve damage caused by transthyretin amyloidosis.

The third stage was from around 2005 to 2015, and was the stage of large-scale discovery of more ncRNAs such as lncRNAs. This is because, around 2005, people began to use genomics and transcriptomics methods such as the Tiling Array to discover and sequence RNAs on a large scale, and found that intergenic DNA regions that we previously thought were untranscribed indeed showed transcription signals, and produced many lncRNAs with a similar appearance to mRNAs—with 5' M^7^G caps and 3' polyA tails. To date, hundreds of thousands of different lncRNAs have been discovered. Later in the 2010s, my group and several other groups discovered a large number of lncRNAs without polyA tails, that is, unconventional lncRNAs. Examples include sno-lncRNAs with small nucleolar RNAs at both ends, and circRNAs. Both are actively studied by our group currently.

**Figure:**
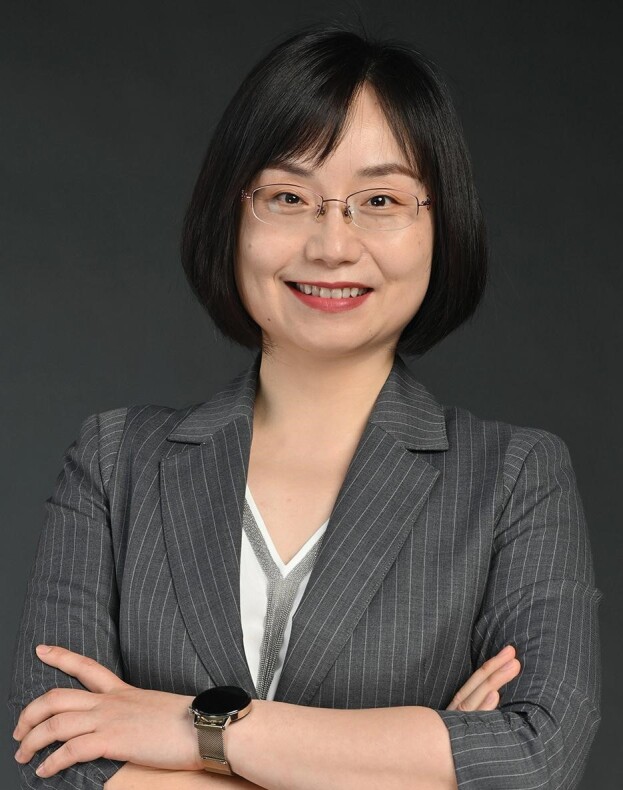
Prof.  Ling-Ling  Chen  has discovered many unconventional lncRNAs.  *(Courtesy of Prof. Chen*)

Moreover, beginning around 2010, people began to pay great attention to the biological functions of lncRNAs. For all kinds of lncRNA species, we try to answer the question of which proteins they interact with in different cellular environments, what conformations they fold into, what physiological roles they play, and what their mechanisms of action are. At present, these questions cannot be explained simply by a general rule, so I think it is currently a challenging and fundamental stage for RNA research.

**Figure 2.**
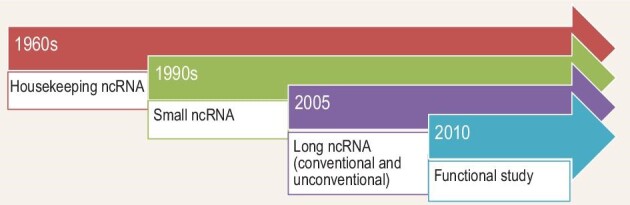
The discovery and research trajectory of ncRNAs.

**Figure 3.**
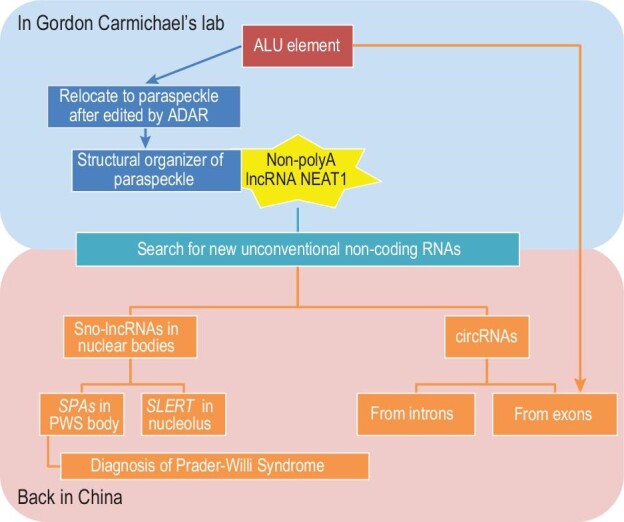
Chen's experience in RNA research.

***NSR*: Will new types of ncRNAs be discovered in the future?**

**Chen:** Yes, I suppose so. According to the length of ncRNAs, we can broadly divide them into long ncRNAs and small ncRNAs, while under these large categories, different RNAs exist in different types of cells, have different ways of carrying out biogenesis, form different conformations and play different roles. I think that in the less-studied cell systems, such as extremophiles, prokaryotes and some cell subtypes in the deep human body, it is very likely that new types of ncRNAs produced by different biogenesis pathways will be discovered.

***NSR*: How did you get into this research area? What are the major contributions you have made to the field?**

**Chen**: There have been some serendipitous moments in my research experience, but actually my research interests have developed in a continuous stream and are traceable. When I became a graduate student in the USA in 2004, my PhD mentor was Prof. Gordon Carmichael, who was a virologist, and my research target was the Alu element, a new research direction of the Carmichael lab. Alu belongs to the short interspersed nuclear elements (SINEs) which uniquely exists in primate genomes and account for 10%–11% of genomes. Many Alus are located in gene-rich regions, and at that time, I asked what the biological consequence of these abundant Alus is. I found that the subcellular location of a subset of mRNAs containing inverted repeated Alus (IRAlus) in their 3’ UTRs would change. Such mRNAs would no longer go out of the nucleus, but stay in a type of subnucleus structure called a paraspeckle. One of the structural organizers of paraspeckles was a lncRNA called NEAT1. This was my initial encounter with ncRNA. More interestingly, of the more than 10 000 lncRNAs that had been discovered at that time, only two did not have polyA tails, and NEAT1 was one of them. So, I wanted to ask: are there other lncRNAs in the transcriptome that don’t have a polyA tail?

I was lucky to receive a research grant of US$200 000 right after I got my PhD in spring 2009. With this grant, I stayed in the Carmichael lab after graduation and performed research semi-independently until 2010. In 2011, I published an article in *Genome Biology*, showing the result of our genome-wide search for non-polyA RNAs in which many non-polyadenylated excised introns and excised exons were found. This project was initiated in Gordon's lab, but finished in my own lab by the end of 2010 at CEMCS, CAS.

Diving into the non-polyA RNAs, in 2012, we reported sno-lncRNAs with small nucleolar RNA ends in *Molecular Cell*. Starting from sno-lncRNAs, we began to study the related nuclear substructures. We have two major directions here: first, we found that some sno-lncRNAs are conspicuously absent in patients with Prada-Willi Syndrome, so the absence of these RNAs can be used as diagnosis markers of this disease; second, we studied the nucleolus-located sno-lncRNA *SLERT*. We not only elucidated how *SLERT* regulates the nucleolus function and structure, but also found a new sub-nucleolus layer, thus expanding the understanding of the nucleolus.

In two articles published in 2013 and 2014, we reported the intron-derived and exon-derived circular RNAs. In collaboration with computational biologists, we found that the circularization mechanism of the exon-derived circRNAs is a pair of inverted orientated complementary Alu elements on the flanking introns next to the future circularizable exon(s); the Alu elements pull the two ends of the distal splice site of exons close and facilitate circularization, and after that, these intronic Alu elements are largely degraded. This is obviously another serendipity: I used to study 3’ UTR Alu elements, so when we met Alu again a few years later, we were able to clarify the production mechanism of this kind of circRNA very quickly. We have now been studying circRNA for 10 years. We have characterized some key features of circRNAs, including their production regulation, conformation, degradation and how their biogenesis would impact innate immunity. We recently also begun to explore their potential applications.

This is my research experience in this field. It has been a very interesting and fulfilling experience for me to start with the discovery of RNA molecules, and then to characterize them, study their functions and see their clinical potential.

## LNCRNA RESEARCH: NO RULES YET, CAUTION NEEDED

***NSR*: What is the importance of ncRNAs in living systems? Are they as important as proteins?**

**Chen:** First of all, housekeeping ncRNAs are definitely very important. They are essential for basic life activities. While most of the other ncRNAs are somewhat regulatory, therefore, compared with proteins, I have to say that protein is essential, but the regulatory roles of ncRNAs are also indispensable.

Nonetheless, regarding the relationship between these two kinds of molecules, I want to say that ncRNAs are often inseparable from proteins—their biogenesis relies upon protein enzymes, and without interacting with proteins and forming complexes with them, lncRNAs cannot fold into functional conformations. On the other hand, many proteins also require lncRNA patterners as ‘chaperons’, ‘scaffolds’, ‘ligands’ and so on to execute proper functions.

***NSR*: Is the folding of RNAs simpler than that of proteins?**

**Chen:** No, the structure of RNAs is more complex than that of proteins. The folding process of proteins roughly follows the thermodynamic conjecture of protein folding, also known as Anfinsen's rule: the primary sequence determines the three-dimensional structure, and the three-dimensional structure determines the function. However, single-stranded RNAs, especially the long single-stranded RNAs, are very flexible. It is easy for them to form knots and difficult for them to fold into functional conformations. So, in cells, the RNAs need to interact with proteins, DNAs and other biomolecules to achieve their folding. Moreover, at different stages of RNA synthesis and functioning, the RNAs may interact with different macromolecules and form different conformations. That is to say, the folding of RNA depends not only on the sequence itself, but also on the multidimensional factors of other molecules in the cells. To date, there is no simple theory that can summarize the folding mechanism of RNAs, and it is difficult to predict their structures with the current knowledge frame.

***NSR*: How much do we understand lncRNAs?**

**Chen:** LncRNAs are highly diversified, and have a role in various biological activities. Unfortunately, their study has been very difficult and to date we still lack a robust way of understanding these molecules. For example, in the early 1990s, a very important lncRNA called Xist was discovered. Xist plays an essential role in the random inactivation of sex chromosomes in female mammals, and has attracted the attention of many researchers. It is safe to say that before the appearance of the Tiling Array in 2005, it was this particular RNA that sustained the whole field of lncRNA research. But now, more than 30 years later, there is still a lot of argument about its functional mechanisms.

Functional lncRNAs can be as short as several hundred bases, or as long as 36 000 bases—Xist is about 21 000 bases long in humans. The function of the long lncRNAs may depend on some of its key modules for interacting with co-factors, but how do these key modules form? How do they fold alone, and with interaction with proteins? No general mode of action has been found yet.

***NSR*: Compared to linear lncRNAs, what are the characteristics of circRNAs?**

**Chen:** Once circularized, the RNA structure becomes more stable. So, in cells, the degradation and synthesis rate of circRNAs are usually very slow. However, under some physiological or pathological conditions, they can be rapidly degraded, thus the slow production and rapid degradation of these RNA molecules may allow them as a group to play a regulatory role.

Indeed, many relatively short circRNAs, the ones shorter than 500 nucleotides, easily form intra-molecular imperfect double-stranded (ds) regions, thus folding into a class of molecules with special structures to interact with proteins, such as the innate immune factor, the dsRNA-activated protein kinase R. This suggests that circRNAs can be developed into RNA aptamers for biomedical applications.

To date, there is no simple theory that can summarize the folding mechanism of RNAs... we still lack a robust way of understanding these molecules (lncRNAs).

—Ling-Ling Chen

***NSR*: Do we have sufficient research methods for the study of lncRNA and circRNA?**

**Chen:** Researchers have always been increasing the number of research methods for RNA. At present, almost all the research methods for proteins, such as immunoprecipitation, immunofluorescence and structural biology, can be adapted or evolved for RNA studies. But generally speaking, they are not so convenient for RNA as they are for proteins, or the resolution is not as high as for proteins. For circRNAs it's even more challenging, because one needs to distinguish circRNAs from linear RNAs with the same sequences, and in all experiments, a control group of linear RNA is needed.

I think that to do RNA research, on the one hand, we should find the most suitable research methods for the particular RNA we are studying. On the other hand, we need to jump out of the existing research framework to explore new research methods.

***NSR*: Where might ‘the new research methods out of the existing framework’ go?**

**Chen:** Firstly, the folding and structure of RNA is highly flexible and often in a dynamic state, so we need higher resolution and more time-resolving methods to capture its states. How shall we do it? We need to integrate interdisciplinary approaches for RNA functional studies, including RNA-chemistry, RNA-biophysics and RNA-based cell biology ideas, with an ultimate goal of resolving the spatial structures of lncRNA and lncRNA-protein complexes at the single molecule and atomic resolution, providing essential RNA structure–function relationships.

Secondly, we should pay more attention to the *in vivo* state of RNAs and their interacting partners. At present, many studies have been carried out with *in vitro* cultured cell lines, which may be quite different from the cell states *in vivo*.

Moreover, we need to study the physiological function of RNAs in greater detail. RNAs are not highly conserved among species. The function of an RNA molecule may be specific in a certain species and under certain physiological or pathological conditions, so we need to be very careful in our research. Future development of appropriate animal models and human organoids are warranted for lncRNA-related phenotypic analyses, disease models and therapeutic potential *in vivo*.

***NSR*: What advice do you have for researchers in other fields who want to study ncRNAs?**

**Chen:** ncRNAs are indispensable in many aspects of life, and are related to many fields such as cancer, immunity and neuronal systems. Researchers in these fields have begun to work on RNAs or use RNA tools to facilitate their studies. I think the most important thing is that you need to know that RNA has its own features. You need to take the specific RNA you are interested in seriously. This includes clarifying its location on the gene, its biogenesis pathway, its subcellular localization and other basic features before you can choose the appropriate research methods and get convincing and consistent research results.

For an easy example, if one lncRNA is located in the nucleus, if you simply knock it down with RNAi (RNA interference) technology, as you would knock down a protein, the result would be that the RNAi may not enter the nucleus at all, resulting in misleading experimental conclusions.

## THE DAWN OF CLINICAL APPLICATION

***NSR*: There are some lncRNAs and circRNAs that have shown promise for clinical application. Can you give some examples?**

**Chen:** lncRNA and circRNA are involved in many different diseases. They have the potential to be used in the diagnosis and treatment of these diseases.

As a diagnostic biomarker, one example is what I just mentioned: by identifying an absence of several sno-lncRNAs in the Prada-Willi Syndrome patients, optimized PCR (polymerase chain reaction) primers or CIRSPR-Cas12 systems have allowed us to easily detect their absence in Prada-Willi Syndrome dried-blood-spot samples. If pushed to the clinic, we will be able to perform routine screenings in newborns—importantly, if there is early diagnosis and intervention with a growth hormone, children with this genetic disease will have the chance to live a normal life.

For the treatment of diseases, some diseases are caused by the abnormality of lncRNAs with regulatory functions, so supplementation of such RNAs can produce a therapeutic effect. Moreover, we do not need to supplement the complete long RNA, but can turn functional modules of lncRNAs, which may be as short as 20 nucleotides, into RNA aptamer drugs for treatment.

For female scientists... Don't say ‘I quit’ so easily.

—Ling-Ling Chen

Particularly, as circRNA is more stable and more structural than lncRNA with the same length, it is more suitable to act as aptamers. My group has found that circRNA aptamers have the potential to treat diseases with excessive PKR (protein kinase R) activation such as psoriasis, at least in animal models. In addition, what we found that synthesized circRNAs with proper conformation generally lack of immunogenicity will also aid the development of a broader spectrum of the next generation RNA therapeutics.

My thoughts have changed over the years. Five years ago, when our team made a discovery, I might have published the article and moved on to the next topic. But now when we make a discovery, I think one step further about how the particular discovery may be applied and how it can help with people's health. After such considerations, I prefer to have the right collaborators, hoping that they can help our team translate these ideas into potentially practical applications. It is absolutely okay for scientists to do only basic research, but one may think about what comes next and open up the possibilities—that is the value of the research funds invested by the state.

***NSR*: In terms of the biomedical application of scientific discoveries, what do you think China needs to improve?**

**Chen:** I think we still lack various types of talented people on the path from scientific innovation to technological application. On this path, we need not only scientists, but also policy support and social input, so we need experienced science-policy communicators and business operators with an understanding of life sciences.

In the past 10 years, China has invested a lot in the field of ncRNA and trained a group of talented people. I hope that these young people will become the backbone of not only RNA research, but also of RNA innovation and translational research. It would be my pleasure to work with these young people.

## WOMEN IN SCIENCE: FACE THE DIFFICULTIES AND NEVER GIVE UP

***NSR*: What are the social responsibilities of scientists? What would you do yourself?**

**Chen:** I think I am very lucky to live in today's China, which respects knowledge and innovation, so that I can do the scientific research work I want to do and also, luckily, get recognition in the field. In terms of my social responsibilities, the first thing is to do solid work in scientific research. I am 45 years old now. I hope I will not stagnate here, but make new discoveries.

I also hope to lead my research team well and train new talents for the field. As the director of a key laboratory, I will try my best to support young researchers. I will make full use of my research experience in the field of RNA to communicate with them and provide them with enough support, at least in the first several years of their independence, a career stage that needs support the most.

In addition, my team and I will actively participate in science popularization to enhance the public's interest in science, especially children and teenagers. I hope that the young generation will be proud to become scientists in the future.

***NSR*: What advice do you have for young researchers, especially female researchers?**

**Chen:** Doing scientific research is a wonderful thing. You are pursuing the truth, and when you have the opportunity to pursue the truth, your life may reach a scientific wonderland. The word ‘research’, *re*-search, means that it is a tortuous but spiraling upward process. So, I hope that young researchers will persist and not give up when they encounter difficulties, and there will be a harvest in the end.

For junior female scientists, I want to say: do not make the day you get your PhD degree the last day of your scientific career. In the field of life sciences, there are more female graduate students than male, but the proportion of females who stick to the path of scientific research and eventually become professors is very low. This disparity indicates that many female scientists leave active academic research early in their careers.

For female scientists, there are obvious social and family responsibilities as mothers and daughters, but as long as one has the passion for doing research or following another similar career in the STEM fields, one should be self-motivated, self-reliant and persistent. When you encounter difficulties, I hope you will not complain, but actively solve the problems by seeking help from family, friends, institutions and society to get through the most difficult stages. For example, even if you do have to quit in the first two years after giving birth, please do come back after this specific stage. Don’t say ‘I quit’ so easily.

As a mid-career female scientist, I also frequently face difficulties with regard to balancing my roles as a team leader, a mother, a daughter and a wife. In a similar way, never say ‘I quit’, and be smart in life and persistent in your career.

## Notes

Weijie Zhao is an NSR news editor based in Beijing, China.

